# Using cardiac magnetic resonance and computational modelling to assess the systemic right ventricle following different Norwood procedures: a dual centre study

**DOI:** 10.1186/1532-429X-17-S1-M12

**Published:** 2015-02-03

**Authors:** James Wong, Pablo Lamata, Rahul H Rathod, Sophie Bertaud, Nathalie Dedieu, Hannah Bellsham-Revell, Kuberan Pushparajah, Reza Razavi, Tarique Hussain, Tobias Schaeffter, Tal Geva, Gerald F Greil

**Affiliations:** 1 KCL, London, UK; 2 Boston Children's Hospital, Boston, MA, USA

## Background

The Norwood procedure for hypoplastic left heart syndrome (HLHS) is performed either via a right ventricle to pulmonary artery (RVPA) shunt or a modified Blalock Taussig (MBT) shunt. A ventriculotomy is used to insert the RVPA shunt and results in a scar on the right ventricle (RV). The affects on ventricular shape and function are assessed

## Methods

A retrospective analysis of 93 cardiac magnetic resonance scans in subjects with HLHS was performed (59 MBT shunt, 34 RVPA shunt) incorporating data from two congenital centres at varying stages of surgery. Longitudinal and short axis cine images were used to create a computational cardiac atlas and global strain was assessed using Feature Tracking analysis.

## Results

**Figure 1:**
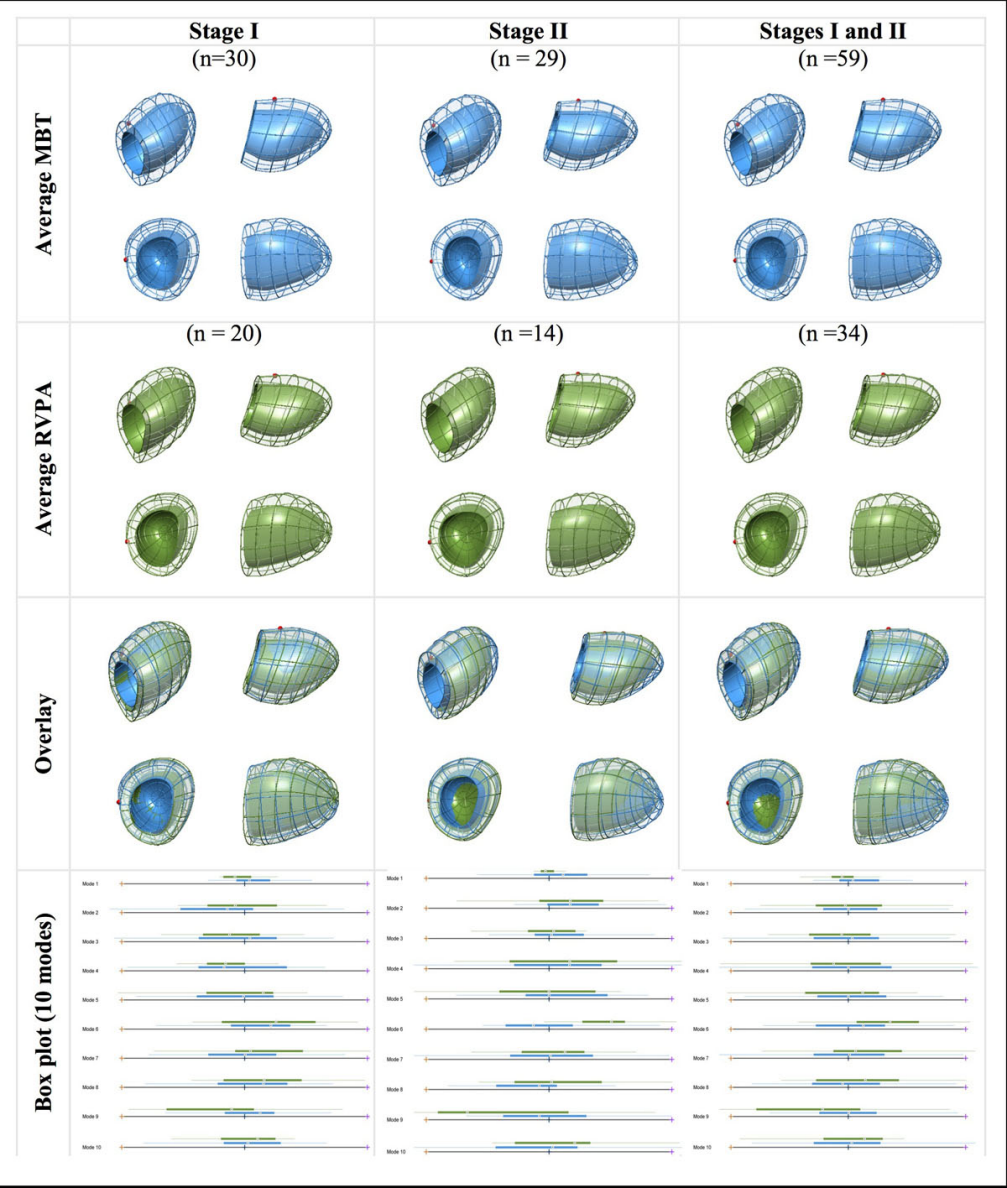


Figure 1 shows the results of the three dimensional shape analysis. Those receiving a RVPA shunt had highly significant differences (*p* <0.00001) in remodelling of the RV corresponding to ventricular dilatation (p = 0.001) and increased sphericity (*p* = 0.006). Differences were evident only after scarring had occurred. Despite preserved ejection fraction in both groups, in the RVPA shunt group functional strain was reduced across multiple ventricular axes including: reduced systolic longitudinal function (strain rate *p* < 0.0001); reduced diastolic longitudinal function (strain rate *p* = 0.0001); and reduced midventricular systolic circumferential function (strain p < 0.0001).

## Conclusions

Computational modelling tools reveal subtle differences in ventricular remodelling in those with HLHS undergoing a RVPA shunt with focal scarring leading to altered functional markers of strain. Although comparative early outcome data shows no differences in survival the need for continued surveillance is warranted, as effects may not become apparent until later years.

## Funding

None.

